# Baicalein Interactions with Lipid Membrane Models: Implications for Its Protective Role against Respiratory Viral Infections

**DOI:** 10.1021/acs.langmuir.5c00161

**Published:** 2025-04-07

**Authors:** Bruna Alves Martins, Giovanna Eller Silva Sousa, Alexandre Mendes de Almeida, Karina Alves Toledo, Osvaldo N. Oliveira, Sabrina Alessio Camacho, Pedro Henrique Benites Aoki

**Affiliations:** † School of Sciences, Humanities and Languages, São Paulo State University (UNESP), Assis, SP 19806-900, Brazil; ‡ Institute of Biosciences, Letters and Exact Sciences, São Paulo State University (UNESP), São José do Rio Preto 15054-000, Brazil; § São Carlos Institute of Physics, University of Sao Paulo (USP), São Carlos, SP 13566-590, Brazil

## Abstract

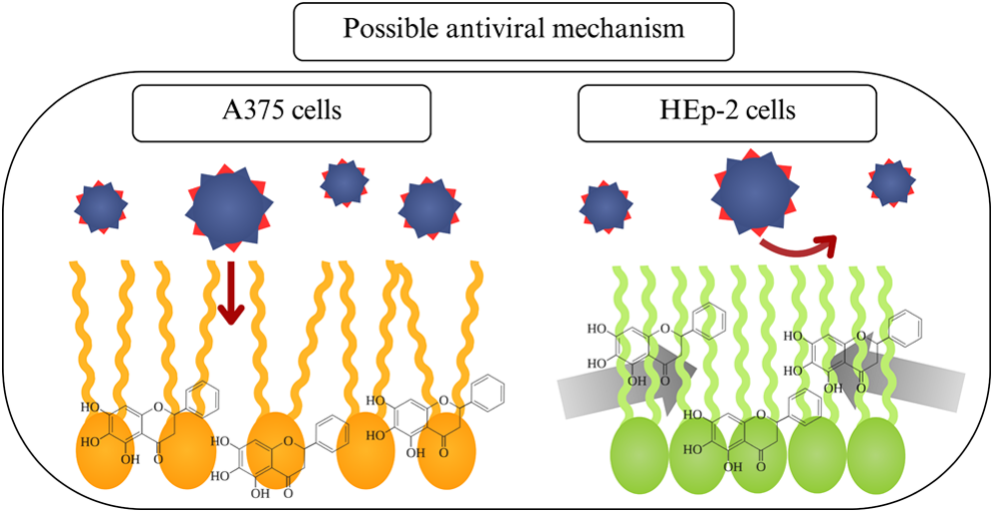

Flavonoids are known for their antioxidant, anti-inflammatory, antitumoral, and antiviral properties, as is the case for baicalein derived from the roots of *Scutellaria baicalensis*, which is effective against respiratory viral infections. In this study, we investigate the molecular mechanisms underlying the interaction between baicalein and Langmuir monolayers as models for cell membranes. For comparison, we analyzed monolayers from lipid extracts of two cell lines: oropharyngeal carcinoma (HEp-2), which is susceptible to respiratory viral infections, and primary melanoma (A375), which is not. Baicalein incorporation into A375 lipid extract monolayers shifted the π–*A* isotherms to larger areas, reducing monolayer stability. In contrast, its incorporation into HEp-2 lipid extract monolayers shifted the π–*A* isotherms to smaller areas, enhancing both compaction and stability. Polarization-modulation infrared reflection–absorption spectroscopy (PM-IRRAS) revealed that baicalein interactions with A375 lipid extracts involved electrostatic attractions and repulsions with choline and phosphate headgroups, disrupting chain organization and expanding the monolayer. In HEp-2 lipid extracts, baicalein interacted strongly with phosphate headgroups and lipid chains, increasing chain order and stabilizing the monolayer. These findings suggest that baicalein stabilizes HEp-2 lipid membranes, potentially providing a protective mechanism against respiratory viral infections. Its selective interaction with lipid membranes is consistent with its therapeutic potential and role in modulating membrane properties to inhibit viral entry.

## Introduction

Flavonoids are phenolic compounds characterized by aromatic rings and hydroxyl groups, widely recognized for their diverse biological activities, including antioxidant, anti-inflammatory, antineoplastic, antibacterial, antifungal, and antiviral properties. These features made flavonoids the focus of extensive research over the past decades.^1−4^ To date, more than 5000 flavonoids have been identified,^5^ most of which are derived from plant sources. Among them, baicalein, an aglycone compound extracted from the roots of *Scutellaria baicalensis*,^6^ has been utilized in traditional Chinese medicine for treating respiratory tract infections, inflammation, fever, hypertension, and tumors.^7−12^ Baicalein has demonstrated efficacy in inhibiting viral infections such as human respiratory syncytial virus,^13^ Chikungunya virus, and Zika virus.^5^ Its antiviral mechanism involves targeting membrane proteins essential for viral replication, promoting β interferon activation, reducing the phosphorylation of inflammatory pathways, and inhibiting the activation of protein receptors.^13−15^ The molecular mechanisms underlying baicalein uptake via incorporation into the plasma membrane, which may prevent viral binding and subsequent infection, remain unclear.^16−19^ Addressing this knowledge gap is essential to elucidate its cellular protective mechanisms against viral entry and optimize its antiviral potential.^20,21^

The plasma membrane is primarily composed of a complex lipid bilayer with embedded proteins and carbohydrates, functioning as a selective barrier that separates the extracellular environment from the intracellular content and regulates the transport of substances.^22^ Studying the molecular interactions of various substances with this structure is challenging due to the diversity of lipid compositions, which can vary between the inner and outer leaflets as well as across different cell types and tissues.^23^ For instance, some cells exhibit greater permeability to specific molecules or higher susceptibility to bacterial and viral infections compared to others within the same organism.^24^ To understand the therapeutic potential of baicalein against respiratory viral infections, we investigated its molecular incorporation and effects on cell lipid extract monolayers of a lineage permissive to viral replication in respiratory tract infections^21,25^ (HEp-2, derived from oropharyngeal carcinoma)^26^ and compared these effects to those observed in a nonpermissive cell line (A375, derived from melanoma).^27^ The experiments were conducted with cancer cell lines (A375 and HEp-2) because of their easy management in cell culture and rapid capacity of replication compared to healthy cells, providing more reproducibility for the trials.^28,29^ This approach is crucial to identifying the binding sites of baicalein and its incorporation within the cell membrane, which acts as the first barrier of viral infection.

Most studies on the antiviral activity of flavonoids, particularly baicalein, focus on *in vitro* experiments to determine whether the molecule exhibits this bioactivity.^18^ However, the specific cellular mechanisms involved and how the flavonoid triggers the immune response remain largely unknown. Given the complexity and diversity of the plasma membrane, *in vivo* experiments do not allow for precise evaluation of how incorporation of baicalein into the plasma membrane exerts a protective mechanism against respiratory viral infections. In this context, Langmuir films have proven to be efficient models for mimicking cell membranes. By producing a lipid monolayer at the air/subphase interface, it is possible to simulate the interactions involving one-half of the cell membrane lipid bilayer. The Langmuir technique enables precise control over the organization of amphiphilic molecules, such as membrane phospholipids, providing insights into membrane fluidity and molecular packing comparable to the biological cell membrane.^30^ Langmuir monolayers are indeed recognized as effective models for studying molecular interactions between lipid compounds and bioactive compounds, including flavonoids.^31−38^ In this scenario, this study focused on determining the molecular interactions and effects of baicalein on Langmuir membrane models^39^ derived from HEp-2 and A375 cell extracts^34,35^ and exploring how these findings can elucidate the protective action of baicalein against respiratory viral infection by preventing viral permeabilization of the cell membrane. Evidence of baicalein interaction was observed through surface pressure (π-A) isotherms and surface compressibility modulus (C_s_^–1^), further supported by monolayer stability and polarization-modulation infrared reflection–absorption spectroscopy (PM-IRRAS).

## Experimental Section

### Materials and Solutions

The phospholipid 1,2-dihexadecanoyl-*sn*-glycero-3-phosphocholine (DPPC, C_40_H_80_NO_8_P, MW = 734.04 g/mol, 98%) was purchased from Avant Polar Lipid. The flavonoid 5,6,7-trihydroxyflavone (baicalein, C_15_H_10_O_5_, MW = 270.24 g/mol, 98%) was acquired from Sigma-Aldrich, which also applies to the solvents dimethylformamide (DMF, HCON(CH_3_)_2_, MW = 73.09 g/mol, >99%), chloroform (CHCl_3_, MW = 119.38 g/mol, >99%), and phosphate-buffered saline (PBS, pH = 7.4). All materials were used without further purification. Ultrapure water (resistivity = 18.2 MΩ·cm, pH = 5.8) was sourced from a Milli-Q purification system (model Direct-Q 3UV). PBS solution was prepared by dissolving the powder in ultrapure water and used as a subphase in the Langmuir film experiments. A 1 mM DPPC solution was prepared in chloroform, while a 1 mM baicalein solution was first dissolved in DMF and subsequently diluted with chloroform in a 1:3 volumetric ratio (DMF:CHCl_3_). Lipid extract solutions were prepared as described previously,^32^ from primary melanoma (A375, ref0278) and oropharyngeal carcinoma (HEp-2, ref0101), both obtained from Banco de Células do Rio de Janeiro (Rio de Janeiro, RJ, Brazil). Briefly, A375 and HEp-2 cells were detached from culture T-flasks, transferred to Falcon tubes, and centrifuged at 6000 rpm for 5 min. After discarding the supernatants, the resulting cell pellets were resuspended in 1 mL of ultrapure water and vortexed for 10 min. Subsequently, 4 mL of chloroform was added to each tube, followed by another 10 min of vortex stirring. The mixtures were sonicated for 30 min and then centrifugated at 6000 rpm for 10 min, resulting in three phases: an upper aqueous phase containing water-soluble cell fragments, a middle layer with cell mass, and a lower phase enriched with chloroform-soluble fragments.^31,32^ The chloroform-based solutions were transferred to amber vials and stored for further use in Langmuir film experiments. These chloroform-based solutions were designated as A375 and HEp-2 cell lipid extracts as they are enriched with cellular lipids.

### Langmuir Films: Fabrication and Characterization

Langmuir films of neat DPPC, A375, and HEp-2 cell lipid extracts were prepared using a Langmuir trough (KSV-NIMA/KN 2002), with PBS as subphase. The temperature was maintained at 21 °C using a thermostatic bath (SolidShell SSDu–10 L) connected to the Langmuir trough. Different volumes of the baicalein solution were cospread with the lipid solutions to achieve 1:5, 1:2, 1:1, and 2:1 volumetric ratios (baicalein:lipid). These volumetric ratios were selected to observe the maximum effect of baicalein in the lipid extract monolayers, as the interactions investigated in the characterization of Langmuir monolayers occur at molecular levels. The surface pressure versus area (π–*A*) isotherms were recorded by spreading the chloroform lipid solutions onto the air-PBS interface and measuring the surface pressure using a platinum Wilhelmy sensor.^40^ The solvent was allowed to evaporate completely for 10 min, leaving only the lipid or flavonoid molecules at the interface. The molecules were then symmetrically compressed using movable barriers at a constant rate of 5 mm/min.^41^ Each experiment was performed in triplicate, and reproducibility was confirmed with a variability of ±2 mN/m.

Relative area displacements (Δ_RA_) were determined from the π–*A* isotherms using the formula [(*A* – *A*_0_)/*A*_0_] × 100, where *A*_0_ is the extrapolated area at 30 mN/m surface pressure for the monolayers of DPPC and the A375 and HEp-2 lipid extracts without baicalein and *A* is the area with cospread baicalein. The surface compressibility modulus (*C*_s_^–1^), indicative of monolayer elasticity, was determined using the expression *C*_s_^–1^ = −*A*(∂π/∂*A*).^42^ Surface area stability of the monolayers was also examined at a constant surface pressure of 30 mN/m over a period of 100 min, comparing the lipid films with and without baicalein. Surface area stability experiments were conducted for 100 min, as this duration was sufficient to assess variations in monolayer stability while ensuring good reproducibility. This time frame was chosen considering the relative surface area constraints that limit the experimental procedure, such as the maximum barrier closure or opening required to maintain the surface pressure at 30 mN/m. The relative area displacements and stability curves were performed at a surface pressure of 30 mN/m based on the literature, according to which the lateral pressure in the plasma membranes of eukaryotic cells is around 30–35 mN/m.^30,43^ Polarization-modulation infrared reflection–absorption spectroscopy (PM-IRRAS) measurements were performed in a KSV PM1550 (KSV, Finland) Langmuir trough with an incidence angle of 81° and a resolution of 8 cm^–1^. Spectra were collected for Langmuir films of DPPC and the A375 and HEp-2 lipid extracts, both with and without cospread baicalein, using a PBS subphase at a surface pressure of 30 mN/m. Spectral reproducibility was confirmed to ensure that changes observed in the PM-IRRAS could be attributed to baicalein incorporation, considering significant those that exceeded the 8 cm^–1^ equipment resolution.^42^

## Results and Discussion

### Baicalein Incorporation into Langmuir Films of Cell Lipid Extracts

**Figure 1:**
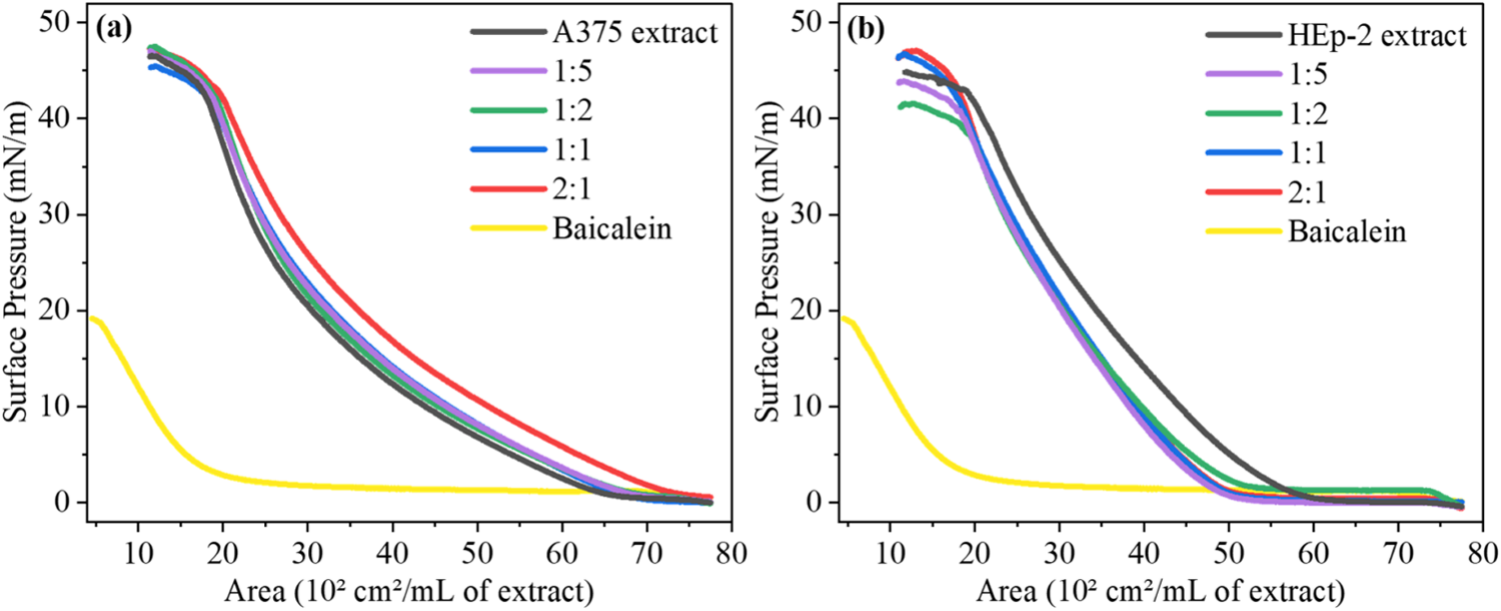
π–*A* isotherms of (a) A375 and (b) HEp-2 lipid extract monolayers cospread with baicalein at different volumetric ratios (baicalein:lipid = 1:5, 1:2, 1:1, and 2:1).

The π–*A* isotherms of A375 and HEp-2 lipid extracts are shown in Figure 1a,b, respectively, including in the presence of different volumetric ratios of cospread baicalein (1:5, 1:2, 1:1, 2:1 baicalein:lipid). These volumetric ratios were selected to ensure the maximum effect (potential) of baicalein in the lipid extract monolayers. Baicalein is surface active on a PBS subphase due to its amphiphilic molecular structure typical of flavonoids like quercetin and myricetin.^44,45^ However, its capacity to form liquid-condensed phases is limited owing to the absence of long hydrophobic tails, and the maximum surface pressure reached in the isotherm is only ∼20 mN/m. For A375 lipid extract monolayers, increasing baicalein ratios shifted the π–*A* isotherms to larger areas, with the most notable effect at a 2:1 ratio, yielding a 21.1% ± 0.9% area increase (Table 1). In contrast, baicalein incorporation into HEp-2 lipid monolayers shifted the isotherms to smaller areas, with negligible changes in the lipid molecule arrangement, indicating limited baicalein integration (Table 1). Subsidiary experiments with the DPPC monolayers, a simplified membrane model, as shown in Figure S1, indicated that baicalein incorporation has effects similar to those on A375 lipid extract monolayers. As shown in Table 1, baicalein induced a shift to larger areas for DPPC. These findings suggest baicalein incorporates into A375 monolayers in a manner potentially driven by interactions with phosphatidylcholine (PC), the most abundant phospholipid in cell membranes.^46^

**Table 1:** Relative Area Displacements Induced by Baicalein Incorporation into A375 and HEp-2 Lipid Extracts, As Well As DPPC Monolayers, at Varying Volumetric Ratios of Baicalein Cospread with Lipid Molecules^a^,^b^

	relative area displacements (%)		
	A375	HEp-2	DPPC
volumetric ratio (baicalein:lipid)	*A*_0_ = 38.6	*A*_0_ = 49.1	*A*_0_ = 46.5
(1:5)	*A* = 39.4 ± 0.8	*A* = 41.8 ± 2.2	*A* = 50.6 ± 1.3
	Δ_RA_ = 2.1% ± 2.2%^a^	Δ_RA_ = −14.9% ± 3.6%^b^	Δ_RA_ = 8.6% ± 2.7%^b^
(1:2)	*A* = 37.8 ± 1.2	*A* = 42.7 ± 1.4	*A* = 53.9 ± 1.5
	Δ_RA_ = 2.0% ± 3.2%^b^	Δ_RA_ = −13.0% ± 2.4%^a^	Δ_RA_ = 15.9% ± 3.1%^b^
(1:1)	*A* = 41.6 ± 1.7	*A* = 42.7 ± 1.7	*A* = 52.0 ± 2.3
	Δ_RA_ = 7.7% ± 4.3%^ab^	Δ_RA_ = −13.1% ± 3.5%^b^	Δ_RA_ = 11.7% ± 5.0%^b^
(2:1)	*A* = 46.7 ± 0.3	*A* = 39.0 ± 1.6	*A* = 69.6 ± 2.3
	Δ_RA_ = 21.0% ± 0.9%^a^	Δ_RA_ = −20.6% ± 3.3%^b^	Δ_RA_ = 49.4% ± 4.9%^a^

a The relative area displacements (Δ_RA_ = [(*A* – *A*_0_)/*A*_0_] × 100) were determined from isotherm data, where *A* and *A*_0_ represent the extrapolated areas to zero pressure taken at a surface pressure of 30 mN/m for monolayers with and without baicalein, respectively. Values are given in 10^2^ cm^2^/mL for lipid extracts and Å^2^ for DPPC.

b Statistical differences are indicated by different lowercase letters within the rows, indicating a significant difference according to the Tukey test (*p* < 0.05).

**Figure 2:**
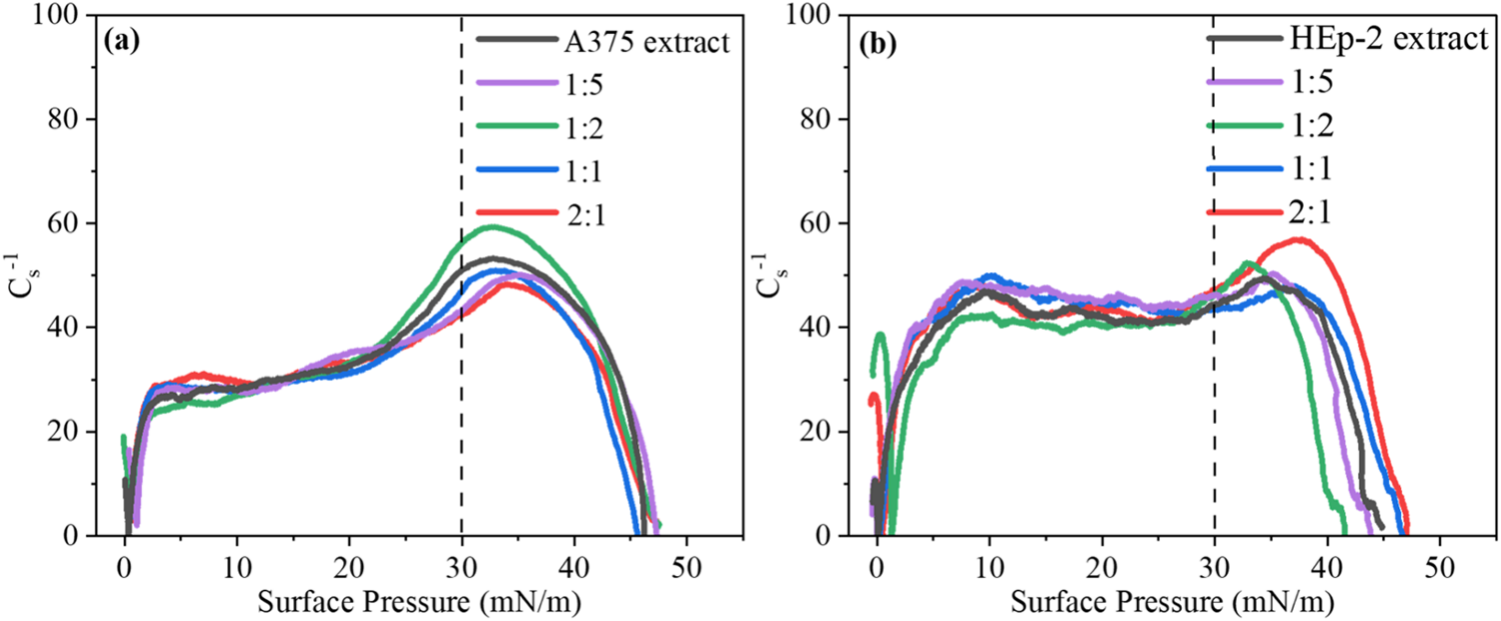
Surface compressibility modulus (*C*_s_^–1^) of (a) A375 and (b) HEp-2 lipid extract monolayers cospread with baicalein at different volumetric ratios (baicalein:lipid = 1:5, 1:2, 1:1, and 2:1).

Baicalein incorporation into A375 lipid extract monolayers caused slight variations in the compressional modulus (*C*_s_^–1^) at 30 mN/m in Figure 2a. *C*_s_^–1^ values decreased from 51 to 43, 47, and 42 mN/m for 1:5, 1:1, and 2:1 (baicalein:lipid) volumetric ratios, respectively, with an increase to 56 mN/m observed only at the 1:2 ratio. These results indicate that A375 monolayers generally became more flexible in the presence of baicalein. These *C*_s_^–1^ values are typical of a liquid-expanded phase, with no evidence of a phase transition induced by baicalein incorporation into A375 lipid extract monolayers.^47,48^ As for the HEp-2 lipid extract monolayer, Figure 2b shows almost no effect on *C*_s_^–1^ upon cospreading baicalein at any volumetric ratio. Therefore, baicalein incorporation neither altered membrane elasticity nor induced a phase transition, maintaining the HEp-2 monolayers in the liquid-expanded phase. For comparison, the neat DPPC monolayer exhibited a substantial reduction in *C*_s_^–1^ values at 30 mN/m upon baicalein incorporation (Figure S1b). Cospreading baicalein at a 1:1 volumetric ratio reduced *C*_s_^–1^ from 147 to 105 mN/m, indicating increased fluidity and disruption of the tightly packed DPPC monolayer.^45^ Similarly to the A375 lipid extract monolayer, this enhanced fluidity did not induce a phase transition from the liquid-condensed phase.^49,50^

### Stability of the Langmuir Films in the Presence of Baicalein

**Figure 3:**
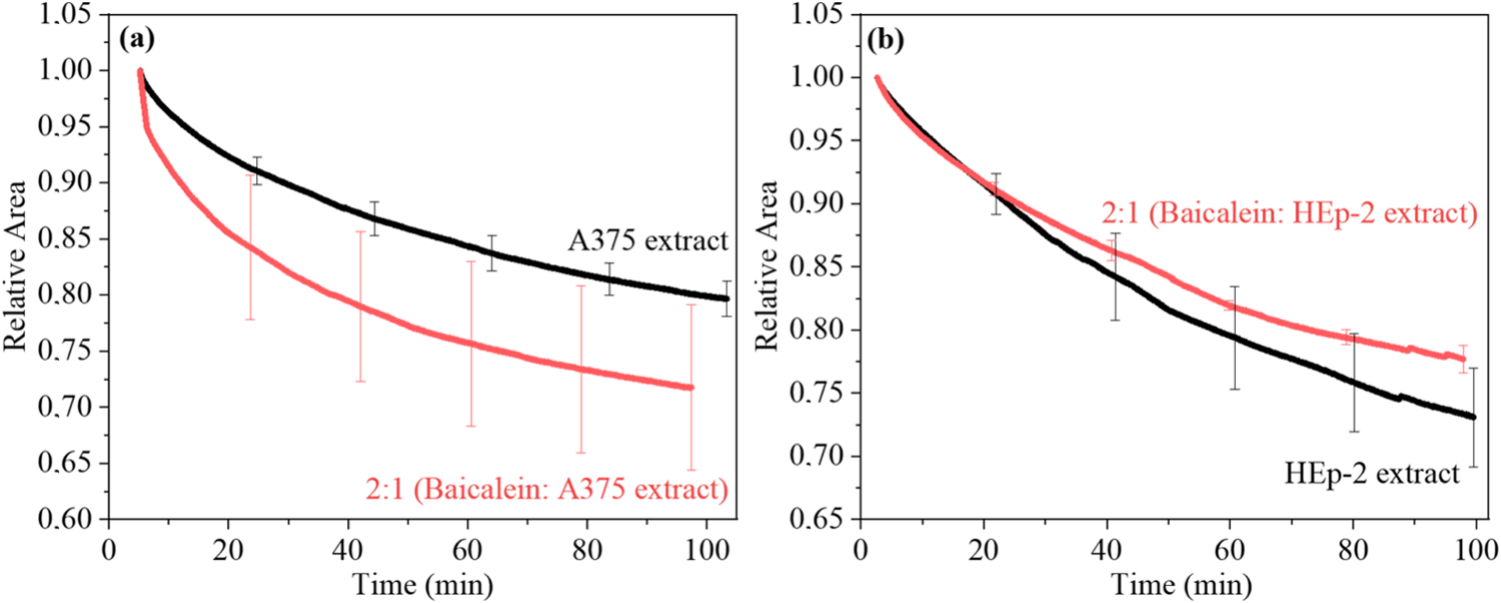
Relative area versus time over 100 min for (a) A375 and (b) HEp-2 lipid extract monolayers on PBS subphase and cospread with baicalein at 2:1 volumetric ratio (baicalein:lipid). The error bars represent the standard deviations of triplicate measurements (stability curves) conducted for each monolayer.

The stability of the Langmuir films was evaluated by tracking relative area changes over time at a constant surface pressure of 30 mN/m. Figure 3a,b shows the stability profiles of A375 and HEp-2 lipid extract monolayers cospread with baicalein at a 2:1 (baicalein:lipid) volumetric ratio. For comparison, Figure S1c presents the stability curves of neat DPPC monolayers on a PBS subphase and cospread with baicalein at a 1:1 ratio. In the absence of baicalein, A375 and HEp-2 lipid extract monolayers exhibited a decrease in relative area, attributed to the oxidative degradation of unsaturated lipid chains by reactive oxygen species (ROS) in the environment, leading to material loss to the subphase.^51^ By contrast, neat DPPC monolayers displayed high stability on the PBS subphase, with relative area trends typical of saturated lipid systems.^38^ Incorporation of baicalein at a 2:1 ratio caused an approximately 7% reduction in the relative area of the A375 lipid extract monolayer over 100 min, indicating decreased stability. Similarly, neat DPPC monolayers cospread with baicalein at a 1:1 volumetric ratio (baicalein:DPPC) exhibited a comparable ca. 2% reduction in relative area (Figure S1c). These findings suggest that baicalein destabilizes A375 and DPPC monolayers, likely facilitating material loss from the interface to the subphase.^51^ On the other hand, baicalein incorporation into the HEp-2 lipid extract monolayer at the same 2:1 volumetric ratio resulted in ca. 3% increase in relative area (Figure 3b), indicating enhanced monolayer stability. A similar stabilizing effect was reported for DOPC monolayers cospread with 10 μM quercetin due to hydrogen bonding between flavonoid molecules and lipid monolayers.^19^ The stabilization of the HEp-2 lipid extract monolayer by baicalein is consistent with the membrane stabilization reported as a key mechanism in the protective biological activity of flavonoids.^52−54^ This may have important biological implications for the role of baicalein, as we shall comment upon while discussing the PM-IRRAS results.

### Molecular Interactions between the Langmuir Films and Baicalein

**Figure 4:**
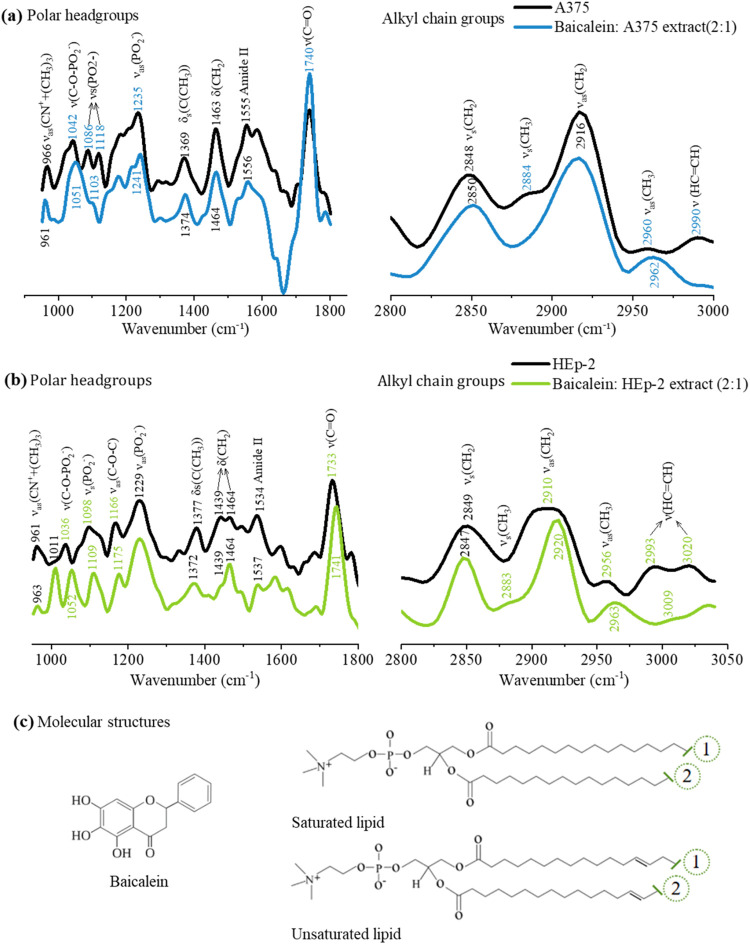
PM-IRRAS spectra recorded for (a) A375 and (b) HEp-2 lipid extract monolayers in the absence and in the presence of cospreading baicalein at 2:1 volumetric ratio (baicalein:lipid) and constant surface pressure of 30 mN/m. The left panels highlight bands related to polar headgroups, while the right panels refer to those associated with alkyl chain groups. (c) Molecular structures of the flavonoid baicalein and two phosphatidylcholines (one saturated and one unsaturated, differing in chain length, represented as 1 and 2). These phosphatidylcholines are the most abundant lipids in the composition of both cell lipid extracts.

Figure 4 presents the PM-IRRAS spectra for A375 and HEp-2 lipid extract monolayers, both without and cospread with baicalein at a 2:1 volumetric ratio. The vibrational modes of the polar headgroups and aliphatic chains are shown in the left and right panels, respectively, with the corresponding assignments provided in Table 2. For the A375 lipid extract monolayer in Figure 4a, ν_as_(CN^+^(CH_3_)_3_) shifted from 966 to 961 cm^–1^ upon baicalein incorporation. Notable changes were also observed in phosphate group vibrations: ν(C–O–PO_2_^–^) at 1042 cm^–1^ shifted to 1051 cm^–1^, and ν_s_(PO_2_^–^) at 1086 and 1118 cm^–1^ merged into a shoulder at 1103 cm^–1^.^37^ These modifications suggest attractive interactions between baicalein and choline groups and repulsive interactions with phosphate groups. At the pH of the PBS subphase (7.4), baicalein is negatively charged (p*K*_a1_ = 5.5),^55,56^ enabling these electrostatic interactions. The carbonyl ester group (ν(C=O)) at 1740 cm^–1^ also displayed an increased relative intensity, indicating structural changes in the monolayer, such as dehydration.^37^ For DPPC monolayers cospread with baicalein in Figure S2, the choline mode shifted from 975 to 963 cm^–1^. Significant alterations were also noted in the phosphate group vibrations: the ν(C–O–PO_2_^–^) band at 1054 cm^–1^ disappeared, and the ν_as_(PO_2_^–^) band shifted to 1228 cm^–1^ with broadening, reflecting changes in phosphate group orientation.^57^ Additionally, the ν(C=O) band shifted from 1743 to 1737 cm^–1^, indicating increased monolayer hydration.^37^ These results align with previous studies on DPPC monolayers and quercetin,^45^ which highlighted electrostatic interactions with lipid headgroups, particularly ν_as_(PO_2_^–^) and ν(C=O). Ferreira et al.^45^ suggested that such interactions could account for the increased area in π–*A* isotherms and potentially enhance flavonoid incorporation into cell membranes. Minimal changes were observed in the aliphatic chains of the A375 lipid extract monolayer upon baicalein incorporation. The intensity ratio (IR) between ν_s_(CH_2_) and ν_as_(CH_2_) increased slightly from 0.53 to 0.60, suggesting a decrease in the conformational order of the monolayer,^58^ consistent with the observed shift in the π–*A* isotherms to larger area values. Similarly, the DPPC monolayer cospread with baicalein exhibited a minor increase in the IR of the CH_2_ bands from 0.56 to 0.58, also indicating reduced lipid chain ordering.

**Table 2:** Assignments of the Main Vibrational Modes of A375 and HEp-2 Lipid Extract Monolayers along with the Displacement Induced by Baicalein Incorporation at a 2:1 Volumetric Ratio

	A375 lipid extract (cm^–1^)		HEp-2 lipid extract (cm^–1^)		
assignments	PBS	baicalein cospread	PBS	baicalein cospread	refs
ν(HC=CH)	2990		2993 and 3020	3009	(33,35)
ν_as_(CH_3_)	2960	2962	2956	2963	(33)
ν_as_(CH_2_)	2916	2916	2010	2920	(64,65)
ν_s_(CH_3_)	2884			2883	(33)
ν_s_(CH_2_)	2848	2850	2849	2847	(33,64)
ν(C=O)	1738	1740	1733	1741	(33)
amide II	1555	1556	1534	1537	(66,67)
δ(CH_2_)	1463	1464	1439 and 1464	1439 and 1464	(33,64,68)
ν (CH_2_)	1369	1374	1377	1372	(31,68)
ν_as_(PO_2_^–^)	1235	1241	1229	1229	(33)
v_as_(C–O–C)			1166	1175	(34,51,66)
ν_s_(PO_2_^–^)	1086 and 1118	1103	1098	1109	(64,66)
ν(C–O–PO_2_^–^)	1042	1051	1036	1052	(33)
ν_as_(CN^+^ + (CH_3_)_3_)	966	961	961	963	(65,69)

Baicalein incorporation into the HEp-2 lipid extract monolayer shifted ν(C–O–PO_2_^–^) from 1036 to 1052 cm^–1^, and ν_s_(PO_2_^–^) from 1098 to 1109 cm^–1^. Additionally, the relative intensity of ν_s_(PO_2_^–^) increased in comparison to that of ν(C–O–PO_2_^–^), indicating reorganization of the phosphate groups. These changes are consistent with repulsive electrostatic interactions between deprotonated baicalein species and phosphate groups, similar to interactions observed in A375 and DPPC monolayers.^57^ The νas(C–O–C) band shifted from 1166 to 1175 cm^–1^, suggesting rearrangement of ester bonds,^59^ while the ν(C=O) band shifted from 1733 to 1741 cm^–1^, indicating dehydration of the monolayer.^37^ More pronounced effects were observed in the lipid chains of HEp-2 monolayers compared to A375 and DPPC monolayers. The ν_as_(CH_2_) and ν_as_(CH_3_) bands shifted to higher wavenumbers, from 2910 to 2920 cm^–1^ and from 2956 to 2963 cm^–1^, respectively. Additionally, the IR ratio of ν_s_(CH_2_)/ν_as_(CH_2_) decreased from 0.77 to 0.63, indicating a significant increase in lipid chain order compared with the changes observed in A375 and DPPC membranes. The ν(HC=CH) band due to unsaturated sites merged into a single band at 3009 cm^–1^ from 2993 and 3020 cm^–1^,^33,35^ showing that baicalein incorporation impacts even the unsaturated regions of the lipid chains.^33^

**Figure 5:**
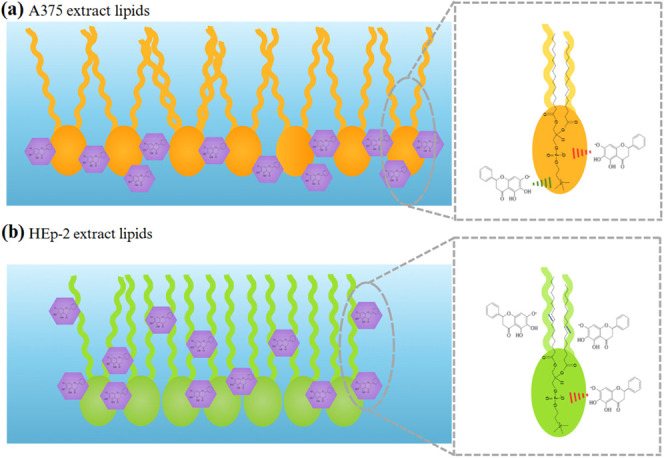
Schematic representation of the interaction mechanisms of baicalein incorporated into (a) A375 and (b) HEp-2 lipid extract monolayers. The insets evidence specific interactions between a PC lipid molecule, the most abundant lipid in both extracts, and baicalein molecules. Green lines indicate attractive electrostatic interactions; red lines show repulsive electrostatic interactions; and dark blue lines illustrate secondary interactions, such as van der Waals interactions.

In summary, baicalein incorporation into A375 lipid extract monolayers appears to be driven by electrostatic interactions with polar headgroups, particularly cholines, phosphates, and carbonyl groups. These interactions slightly disrupt lipid chain order, leading to monolayer expansion and reduced stability, as illustrated in Figure 5a. Baicalein integration into HEp-2 lipid extract monolayers is also mediated by interactions with polar headgroups, including phosphates and carbonyl groups, but it has more pronounced effects on the lipid chains. Baicalein molecules penetrate the aliphatic chains, including in unsaturated regions, resulting in an increased lipid chain order. This enhances monolayer compactness and stability, accompanied by a reduction in monolayer area, as shown in Figure 5b. It should be stressed that this reduction in area could be considered counterintuitive, since when a guest molecule is incorporated into the hydrophobic tails of a Langmuir monolayer, one expects an increase in area. However, if the monolayer is in a liquid-expanded phase, as is the case of HEp-2, such incorporation may occur without this increase. In particular, there may be sufficient space to accommodate the guest molecules while still making the monolayer more compact.

These structural changes align well with the results from the π–*A* isotherms and monolayer stability of the previous section, showing clear differences between A375 and HEp-2 monolayers in terms of baicalein action. Biological implications can be drawn as the stabilizing action by baicalein on the HEp-2 monolayer could be correlated with a protective role for the membrane. Since cell membranes serve as the first barrier to viral entry,^48^ this means that baicalein could have a protective effect against respiratory viral infections by inhibiting viral binding to the plasma membrane. It would be similar to what happens with 27-OH compound in its antiviral activity against SARS-CoV-2.^60^ Also, it could explain the baicalein effect in pre and post treatments of Vero cells in immune assays with SARS-CoV-2,^61^ as survival was enhanced by protecting cells from the virus. This hypothesis is consistent with other studies with baicalein. For instance, the oral administration of baicalein reduced the damage caused in lung tissues on *in vivo* experiments with rats,^61^ and baicalein exhibited antiviral effects on *in vitro* experiments with other viruses as well.^8,62,63^

## Conclusions

This study explored the effects of baicalein incorporation into lipid extract monolayers of HEp-2 (oropharyngeal carcinoma) and A375 (primary melanoma) cells, elucidating the molecular interactions underlying its therapeutic action on cellular plasma membranes of a permissive and a nonpermissive cell line for respiratory viral infections, respectively. π–*A* isotherms revealed that increasing baicalein ratios in A375 lipid extract monolayers caused shifts to larger areas, changing the area by 21.1% ± 0.9% for the 2:1 ratio, indicating greater insertion of baicalein molecules and increased fluidity, as evidenced by changes in the surface compressibility modulus (*C*_s_^–1^). In contrast, baicalein incorporation into HEp-2 lipid extract monolayers displaced the isotherms to lower areas without significant changes as the baicalein ratio increased, suggesting a limited amount of incorporated baicalein molecules. The unaltered *C*_s_^–1^ values further indicated that baicalein does not affect HEp-2 monolayer flexibility. These incorporation mechanisms influenced the monolayer stability: baicalein reduced the relative area of the A375 monolayer by approximately 7%, decreasing its stability, while it increased the relative area of the HEp-2 monolayer by about 3%, enhancing its stability. PM-IRRAS analysis showed significant interactions of baicalein with A375 lipid headgroups, characterized by electrostatic attraction to choline and repulsion from phosphate groups, accompanied by a slight disorganization of the lipid chains. Conversely, in the HEp-2 monolayer, baicalein primarily interacted with the phosphate headgroups via electrostatic repulsion but also penetrated deeper into the lipid tails, inducing pronounced modifications in the aliphatic chains. This deeper penetration was likely facilitated by secondary interactions, even with saturated lipid sites. In summary, these findings highlight the distinct molecular interactions of baicalein with cell membrane models of permissive (HEp-2) and nonpermissive (A375) cell lines for respiratory viral infections. They provide valuable insights into how baicalein may stabilize cell membranes and prevent viral binding, establishing a mechanistic foundation for its potential role in mitigating respiratory viral infections. This also enhances the understanding of *in vitro* systems and guides the development of antiviral applications for flavonoids.

## Supporting Information Available

Additional discussion on baicalein-induced changes into DPPC monolayers, including the π–*A* isotherms, surface compressibility modulus, stability curves, PM-IRRAS spectra, and the assignments of the main vibrational modes for DPPC monolayers (PDF)
