# Supplementary material for: Baicalein Interactions with Lipid Membrane Models: Implications for Its Protective Role against Respiratory Viral Infections
Source: Langmuir. 2025 Apr 7;41(14):9377–85. doi: 10.1021/acs.langmuir.5c00161 (PMC12004914; doi:10.1021/acs.langmuir.5c00161)
Supplement: Supplementary file 1 — la5c00161_si_001.pdf [file la5c00161_si_001.pdf]

Supporting information

Baicalein Interactions with Lipid Membrane Models: Implications for its Protective Role Against Respiratory Viral Infections.

Bruna Alves Martins[1], Giovanna Eller Silva Sousa[1], Alexandre Mendes de Almeida Jr.[1], Karina Alves Toledo[1,2], Osvaldo N. Oliveira Jr[3]; Sabrina Alessio Camacho[1], Pedro Henrique Benites Aoki[1]

[1]São Paulo State University (UNESP), School of Sciences, Humanities and Languages, Assis, SP, 19806-900, Brazil

[2] São Paulo State University (UNESP), Institute of Biosciences, Letters and Exact Sciences, São José do Rio Preto, SP, 15054-000, Brazil

[3]University of Sao Paulo (USP), São Carlos Institute of Physics, São Carlos, SP, 13566-590, Brazil

*Corresponding author: pedro.aoki@unesp.br

Contents:

Figure S1. (a) π-A isotherms and (b) surface compressibility modulus (C_s^{-1}) of neat DPPC monolayer and DPPC monolayers co-spread with baicalein at different volumetric ratios (1:5, 1:2, 1:1, 2:1 baicalein: lipid). (c) Stability curves of neat DPPC monolayer and DPPC monolayer co-spread with baicalein at 1:1 ratio. **S-3**

Figure S2. PM-IRRAS spectra recorded for DPPC monolayers in absence and in presence of co-spreading baicalein at 1:1 volumetric ratio (baicalein: DPPC) and a constant surface pressure of 30 mN/m. The left panel highlights bands related to polar headgroups, while the right panel refers to those associated with alkyl chain groups.

Table S1. Assignments of the main vibrational modes of DPPC monolayers along with the displacement induced by baicalein incorporation at 1:1 volumetric ratio. **S-4**

Contents of the Supporting Information

Number of pages: 6

Number of Tables: 1

Number of Figures: 2

The π-A isotherms of DPPC monolayers in absence and in presence of baicalein, co-spread at different volumetric ratios (1:5, 1:2, 1:1, 2:1 baicalein: DPPC), are displayed in Figure S1a. Neat DPPC isotherm is comparable to those reported in the literature,[1,2] with a molecular area of approximately 54 Å^2 by extrapolating the area from the condensed phase at 30 mN/m to 0 mN/m, and a collapse surface pressure of 56 mN/m. By increasing the baicalein ratio in the co-spread films, the π-A isotherms of DPPC shift toward larger molecular areas, as an indicative of the greater insertion of the flavonoid molecules into the lipid monolayers. The profile of the co-spread isotherms follows that of the neat DPPC isotherm, suggesting that baicalein incorporation disturbs the lipid monolayers without induce a phase transition, even at higher volumetric proportions[3]. Indeed, although baicalein insertion into DPPC films causes a decrease in the C_s^{-1} values at 30 mN/m, from 143 mN/m to 110, 136, 106 and 123 at 1:5, 1:2, 1:1, 2:1 ratio, respectively, the monolayers kept at the liquid-condensed phase (LC) (Figure S1b).[4] This result suggests that the insertion of baicalein causes an increase in the fluidity of the monolayer.

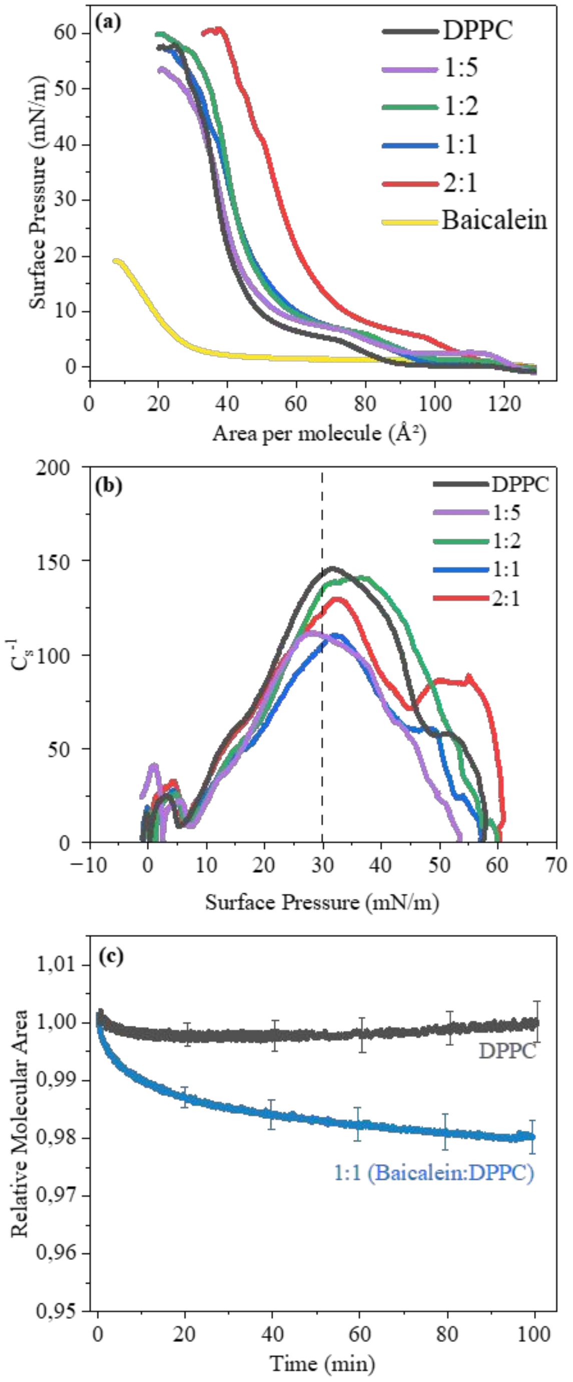

Figure S1. (a) π-A isotherms and (b) surface compressibility modulus (C_s^{-1}) of neat DPPC monolayer and DPPC monolayers co-spread with baicalein at different volumetric ratios (1:5, 1:2, 1:1, 2:1 baicalein: lipid). (c) Stability curves of neat DPPC monolayer and DPPC monolayer co-spread with baicalein at 1:1 ratio.

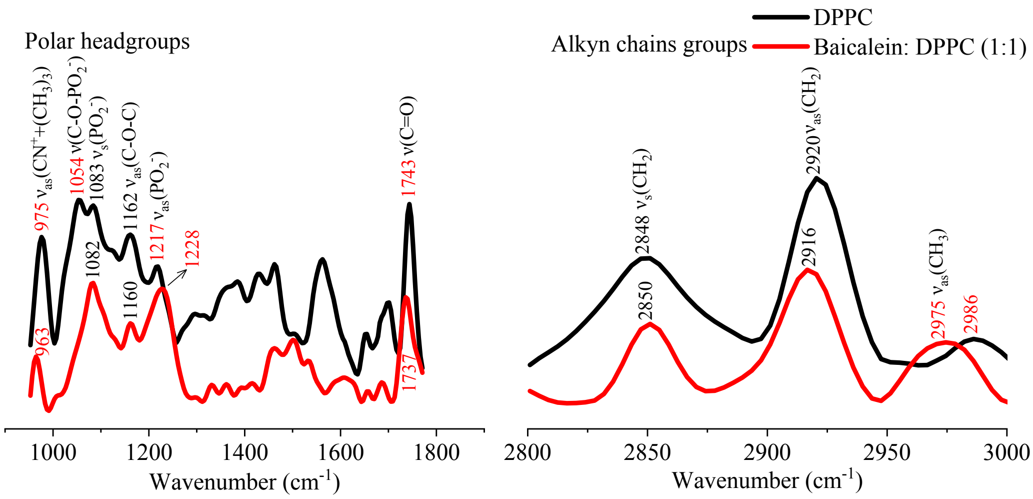

Figure S2. PM-IRRAS spectra recorded for DPPC monolayers in absence and in presence of co-spreading baicalein at 1:1 volumetric ratio (baicalein: DPPC) and a constant surface pressure of 30 mN/m. The left panel highlights bands related to polar headgroups, while the right panel refers to those associated with alkyl chain groups.

Table S1. Assignments of the main vibrational modes of DPPC monolayers along with the displacement induced by baicalein incorporation at 1:1 volumetric ratio.

**Assignments**	**DPPC**		**References**
	Neat	baicalein co-spread	
ν(HC=CH)	-	-	
$\nu_{as}(CH_3)$	2993	2975	[1]
$\nu_{as}(CH_2)$	2920	2916	[2,3]
$\nu_s(CH_2)$	2848	2850	[1,2]
ν(C=O)	1743	1737	[1]
Amide II	-	-	
$\delta(CH_2)$	-	-	
$\nu\ (CH_2)$	-	-	
$\nu_{as}(PO_2^-)$	1217	1228	[1]
ν_{as}(C-O-C)	1162	1160	[4–6]
$\nu_s(PO_2^-)$	1083	1082	[2,4]
$\nu(C\text{-}O\text{-}PO_2^-)$	1054	-	[1]
$\nu_{as}(CN^+ + (CH_3)_3)$	975	963	[3,7]

References

(1) Camacho, S. A.; Kobal, M. B.; Almeida, A. M.; Toledo, K. A.; Oliveira, O. N.; Aoki, P. H. B. Molecular-Level Effects on Cell Membrane Models to Explain the Phototoxicity of Gold Shell-Isolated Nanoparticles to Cancer Cells. *Colloids Surf B Biointerfaces* **2020**, *194*, 111189. https://doi.org/10.1016/J.COLSURFB.2020.111189.

(2) Wei, T. T.; Cao, B. B.; Hao, X. L.; Gu, J. Y.; Wu, R. G. The Interaction of Baicalein with Dipalmitoylphosphatidylcholine Liposomes: Differential Scanning Calorimetry, Synchrotron X-Ray Diffraction, and Fourier Transform Infrared Studies. *Thermochim Acta* **2021**, *703*, 178993. https://doi.org/10.1016/j.tca.2021.178993.

(3) Zhou, Y.; Dong, W.; Ye, J.; Hao, H.; Zhou, J.; Wang, R.; Liu, Y. A Novel Matrix Dispersion Based on Phospholipid Complex for Improving Oral Bioavailability of Baicalein: Preparation, in Vitro and in Vivo Evaluations. *Drug Deliv* **2017**, *24* (1), 720–728. https://doi.org/10.1080/10717544.2017.1311968.

(4) Srisayam, M.; Weerapreeyakul, N.; Barusrux, S.; Tanthanuch, W.; Thumanu, K. Application of FTIR Microspectroscopy for Characterization of Biomolecular Changes in Human Melanoma Cells Treated by Sesamol and Kojic Acid. *J Dermatol Sci* **2014**, *73* (3), 241–250. https://doi.org/10.1016/j.jdermsci.2013.11.002.

(5) Moreira, L. G.; Almeida, A. M.; Nield, T.; Camacho, S. A.; Aoki, P. H. B. Modulating Photochemical Reactions in Langmuir Monolayers of Escherichia Coli Lipid Extract with the Binding Mechanisms of Eosin Decyl Ester and Toluidine Blue-O Photosensitizers. *J Photochem Photobiol B* **2021**, *218*, 112173. https://doi.org/10.1016/j.jphotobiol.2021.112173.

(6) Kobal, M. B.; Camacho, S. A.; Moreira, L. G.; Toledo, K. A.; Tada, D. B.; Aoki, P. H. B. Unveiling the Mechanisms Underlying Photothermal Efficiency of Gold Shell-Isolated Nanoparticles (AuSHINs) on Ductal Mammary Carcinoma Cells (BT-474). *Biophys Chem* **2023**, *300*, 107077. https://doi.org/10.1016/j.bpc.2023.107077.

(7) Pires, F.; Magalhães-Mota, G.; Geraldo, V. P. N.; Ribeiro, P. A.; Oliveira, O. N.; Raposo, M. The Impact of Blue Light in Monolayers Representing Tumorigenic and Nontumorigenic Cell Membranes Containing Epigallocatechin-3-Gallate. *Colloids Surf B Biointerfaces* **2020**, *193*, 111129. https://doi.org/10.1016/j.colsurfb.2020.111129.
